# Design, Simulation and Multi-Objective Optimization of a Micro-Scale Gearbox for a Novel Rotary Peristaltic Pump

**DOI:** 10.3390/mi14112099

**Published:** 2023-11-14

**Authors:** Nikolaos Rogkas, Matthaios Pelekis, Alexandros Manios, Alexandros Anastasiadis, Georgios Vasileiou, Achilleas Tsoukalis, Christos Manopoulos, Vasilios Spitas

**Affiliations:** 1 Laboratory of Machine Design and Dynamics, School of Mechanical Engineering, National Technical University of Athens, Zografos, 15780 Athens, Greece; matheos.p@gmail.com (M.P.); alx.manios@gmail.com (A.M.); alexandros.anastasiadis@epfl.ch (A.A.); gvasileiou@mail.ntua.gr (G.V.); vspitas@mail.ntua.gr (V.S.); 2 Micrel Medical Devices SA, Koropi, 19441 Athens, Greece; achilleas@micrelmed.com; 3 Biofluid Mechanics & Biomedical Technology Laboratory, School of Mechanical Engineering, National Technical University of Athens, Zografos, 15780 Athens, Greece; manopoul@fluid.mech.ntua.gr

**Keywords:** peristaltic pump, drug delivery systems, gearbox, optimization

## Abstract

Peristaltic pumps are widely used in biomedical applications to ensure the safe flow of sterile or medical fluids. They are commonly employed for drug injections, IV fluids, and blood separation (apheresis). These pumps operate through a progressive contraction or expansion along a flexible tube, enabling fluid flow. They are also utilized in industrial applications for sanitary fluid transport, corrosive fluid handling, and novel pharmacological delivery systems. This research provides valuable insights into the selection and optimal design of the powertrain stages for peristaltic pumps implemented in drug delivery systems. The focus of this paper lies in the simulation and optimization of the performance of a power transmission gearbox by examining the energy consumption, sound levels, reliability, and volume as output metrics. The components of the powertrain consist of a helical gear pair for the first stage, a bevel gear pair for the second one, and finally a planetary transmission. Through extensive simulations, the model exhibits promising results, achieving an efficiency of up to 90%. Furthermore, alternative configurations were investigated to optimize the overall performance of the powertrain. This process has been simulated by employing the KISSsoft/KISSsys software package. The findings of this investigation contribute to advancements in the field of biomedical engineering and hold significant potential for improving the efficiency, reliability, and performance of drug delivery mechanisms.

## 1. Introduction

Over the past few decades, micro- and meso-scale pumps have made major contributions in the advancement of delivery systems in the biomedical and biological fields. These devices offer a variety of advantages including their small size and weight, portability, low power consumption, and full control regarding the flow rate range [1]. In particular, peristaltic pumps and peristaltic micropumps (PMP) are essential tools in the biomedical field, mainly used to transport fluids from a lower pressure region towards a higher pressure one. This is achieved via a progressive wave of contraction or expansion waves which propagates along the length of a distensible tube containing fluids [2]. In addition, the most distinct advantage that this type of pump presents over its counterparts is that the transporting fluid is being kept sterile continuously. This is achieved by keeping the fluid contained in the transporting tube, which is compressed in a way that achieves the desired flow rate. Tripathi et al. and Mohith et al. [2,3] have demonstrated these claims by studying the applications of peristaltic micropumps. The former focuses mainly on the peristaltic flow of nanofluids in drug delivery systems by examining the geometry, nanoparticle fraction, temperature, and velocity profiles, as well as the pressure difference across one wavelength during operation. The latter have also classified the various types of peristaltic pumps, alongside their applications.

In this scale, pumps are categorized according to their actuation method. Major actuation methods include electrostatic [4,5], motor [6,7], piezoelectric [8,9,10,11,12], pneumatic [13,14,15,16,17], electrohydrodynamic (EHD) [18,19,20], and thermal [21,22,23] (shape-memory alloys [21], temperature-sensitive hydrogels [22], etc.) actuation, among others [24].

Many researchers have attempted to study the effects of the actuation method on the flow of the working fluid in micropumps. For instance, Mi et al. [25] developed a minimized valveless electromagnetic micropump which can be used as a power source to achieve dynamic culture in organ chips. Chen et al. [12] studied a hydraulic-driven piezoelectric pump with a separable channel for drug delivery (HPPSCDD) to avoid cross-contamination issues. A thermal non-mechanical micropump was investigated theoretically and experimentally by Song and Zhao in 2001 [23]. Berg et al. demonstrated a two-stage discrete peristaltic micropump for microfluidics in which the actuation energy is being provided by an off-wafer compressed nitrogen gas [14]. A very intriguing approach on the actuation methods was studied by Yamatsuta et al. [26] who proposed an alternative concept in the form of a muscle-powered tubular micropump, which provides peristaltic transport by employing *Drosophila melanogaster* larvae on the cell membrane of skeletal muscles to obtain light-responsive muscle tissues. The application of pneumatically driven pumps has also been investigated in the context of Funabot-Suit as described in [27].

However, PMPs also present limitations in given applications, depending on their type. For instance, Forouzandeh et al. have pointed out that single-actuator PMPs (SAPMPs), which are typically pneumatic-driven, are unidirectional by nature. As a result, this could limit their applications where bidirectionality is crucial [24]. Respectively, motor-driven PMPs are challenged in size-constrained applications, such as implantation in small rodents [24]. Electrostatic PMPs, on the other hand, tend to offer excellent miniaturization capabilities and power flow rate efficiency. However, these pumps are limited due to the high operating voltage limit applications due to electrolysis with polar working fluids [24].

One of the most predominant versions of a peristaltic pump, especially in biomedical applications, are roller pumps. The flow in this type of peristaltic pump is generated in a manner that replicates peristalsis in living organisms. This working mechanism further reduces the probability of cross-contamination between the working fluid and any substance that may be contained in the mechanical parts of the pump [28]. The primary benefits of roller pumps include high reliability, simplicity of operation, and cost-effectiveness regarding the disposable tubing [29].

To date, most of the research that has been conducted in the field of peristaltic micro- (or meso-) pumps focuses on simulations of the flow rate and experimental validation. For this purpose, several researchers have developed CFD models and performed FSI (fluid–structure interaction) analysis to study the flow dynamics characteristics [29,30,31,32]. Namely, Manopoulos et al. studied novel tube designs for an infusion roller pump through multiple FSI simulations in order to obtain qualitative results and suppress backflow [29]. J.W Mulholland et al. presented a 2D numerical simulation of a roller pump to assess the possibility of using CFD results in a theoretical blood damage prediction model [30]. Another approach on the matter was made by Elabbasi [32] et al. who investigated the effect of phenomenon such as tube occlusion, tube diameter, and roller speed on the dynamics of the tube using finite element analysis.

A significant aspect of any peristaltic pump lies in its driving mechanism, a subject that has not been adequately investigated and provides space for innovative ideas and novel research. Rhie et al. [33] have taken a novel approach, by designing a screw-driven multi-channel peristaltic pump for portable microfluidic devices. Lee et al. also implemented a novel actuator, with a notched cross-section design that employs less than half the force to fully seal compared to a counterpart with a circular cross-section. This concept makes it possible to create and maintain a low vacuum at low actuation speed [34]. However, despite some studies delving into the transmission mechanisms and actuation methods in micro-peristaltic pumps, there is considerable potential for further exploration in this area.

The mechanisms constituting the peristaltic pump of the current study feature plastic micro-gears. The growing demand for miniaturization in products has given rise to the complexity of such micro-gears, as essential functions increasingly rely on the use of highly precise components [35]. These components are characterized by their low module range of 1–1000 μm, which differentiates them from their macroscale counterparts [36]. The flexibility offered by the nature of their size extends their influence across various industries, including the medical technology among others, such as the watch industry, robotics, aerospace, and automotive engineering [35,37]. The wide applicability of micro-gears also underscores their importance as actuating components within micro-electromechanical systems (MEMS), drawing increasing attention from researchers [38]. The behavior and performance of micro-gears, encompassing load capacity, acoustics, and efficiency, are intrinsically linked to the quality and precision of manufacturing technology, as also demonstrated by their tolerances and surface quality [36]. As these gears are integral to the functionality of critical systems, such as those in medical technology, the demand for stringent quality control strategies has become essential. Miniaturization in manufacturing, epitomized by micro-gears, places unique and demanding requirements on production processes, necessitating innovative approaches to overcome technological limits [39].

This study aims to further explore the vast potential provided by this particular field of research by introducing an innovative approach to incorporate micro-gears into a gearbox designed to operate within this peristaltic pump system The focus of the present work lies mainly in the design, simulation, and multi-objective optimization of the stages of a gearbox, which facilitates the transportation of the working fluid through the flexible tube of a novel rotary peristaltic roller-pump. This concept has been incorporated into the design [40,41,42,43] and has also been studied numerically and experimentally [29,44]. The gearbox consists of two sub-mechanisms. The first component is a powertrain, which is directly connected to a brushless DC motor and its main purpose is the power transmission and speed reduction to the second module, comprised of a planetary gearset. The latter is a disposable part, which is mainly responsible for the transportation of the fluid by compressing and decompressing a flexible tube using a set of rollers, integrated to the planets of the gearset. The main goal of the design is the determination of such machine elements that optimally combine the desired results of four main parameters, which have been selected in accordance with the optimization criteria outlined in [45]. These include adequately low energy consumption and high efficiency of the pump, sufficiently high reliability of the machine elements for each stage, low mechanical noise, as well as a small volume/size of the system.

Previous work regarding micropump actuation indicates that a mechanical actuation via a gearbox has not been yet researched at this scale. The proposed design aims to introduce a proficient and easily implemented mechanism as a new method of micropump actuation. The gearbox for the novel rotary peristaltic pump has been designed and simulated within the framework of the research project Accuflow, which relates to the development of an innovative technological platform for drug delivery devices, designed for combined use with specialized drugs.

## 2. Materials and Methods

This section focuses on the modelling and simulation methods of the system, including the governing equations that were used during the simulation alongside assessing the material selection of the transmitting gears. Τhe transmission mechanism features a multi-stage gearbox where each stage is essential for normal operation due to the need of a high transmission ratio, a change in direction, and space restrictions.

As analysed in [46], different transmission mechanisms are tailored to different applications. Spur and helical gears are mainly used in parallel shafts. The former is mainly utilized due to their simple construction, high efficiency, high precision, and wide range of velocities. The latter also show great prominence, due to the increased efficiency, high load application capacity, higher contact ratio, and quiet operation. Conversely, the applications of straight and spiral bevel gears concern intersecting shafts due to their good precision, suitable for low ratios, high speed, and high load capacity. Spiral bevel gears also contribute to a higher contact ratio and smooth/quiet operation. Hypoid bevel gears, which present lower efficiency and high sliding, are designated for non-intersecting shafts. Another alternative involves worm gear pairs, which are used in skew shafts. Although this configuration often yields lower efficiency, it offers quiet operation, high reduction ratios, and a moderate precision rating. However, their requirement for effective lubrication may raise concerns regarding their suitability for use in such applications. Belt drives also provide notable advantages, showcasing relatively high efficiency, cost effectiveness, and low noise operation. Nonetheless, due to considerations about their shorter service life and their need for regular maintenance they are deemed insufficient.

Transmission mechanisms such as hypoid bevel gears and worm/worm gear pairs, therefore, do not fit the purposes of this research and will not be examined due to their low efficiency. Belt drives would also introduce topological complexity and would ultimately reduce the performance of the system since more stages would be needed in order to achieve the desired topology unless a worm–worm gear pair is used.

### 2.1. Modelling and Simulation of the System

This section presents a comprehensive investigation into the components of the peristaltic pump. The mechanism is constituted of three distinct stages featuring plastic gears. The focus lies on the arrangement of these three discrete stages. The first stage is a helical gear pair followed by the second stage featuring bevel gears. These lead to the disposable part of the mechanism featuring a planetary system stage with two planets, which is required to maintain a speed decrease and thus small and accurate output flow rate. Initially, Computer-Aided Design (CAD) software (SolidWorks 2022, Educational Version) [47] was used for the modeling of these stages. Subsequently, the KISSsoft/KISSsys 2022 software package by KISSsoft AG—A Gleason Company, Bubikon, Switzerland, emerges as the chosen software to perform the simulations. These simulations are anchored in the pursuit of the four distinct optimization goals: increased efficiency, thus lower energy consumption; reduction of the volume; securing the longevity of the components; as well as minimization of mechanical noise. More specifically, it has been proven that the mechanical noise produced from gear meshing is directly associated with the transmission error [48]. Consequently, the noise reduction subject translates to the minimization of the transmission error. This research employs an integrated approach that incorporates modeling, simulations, optimization, and optimal design selection techniques to evaluate the relationship between gear configurations and performance metrics, operating within the defined set of operational parameters.

The optimization aim is to improve the power consumption, which has originally been approximated to be about 0.4 mWh/mL, at a flow rate of 1000 mL/h. To this end, guiding technical specifications have been set for each gearbox stage. These are indicatively described in Table 1, which consecutively ensure the pump’s operation at a system operating power of 0.5 W.

The magnitude orders of the presented values have been specified from the design process of the system, also taking into consideration previous designs of the mechanism [40,41,42,43].

#### 2.1.1. CAD Modelling

**Figure 1:**
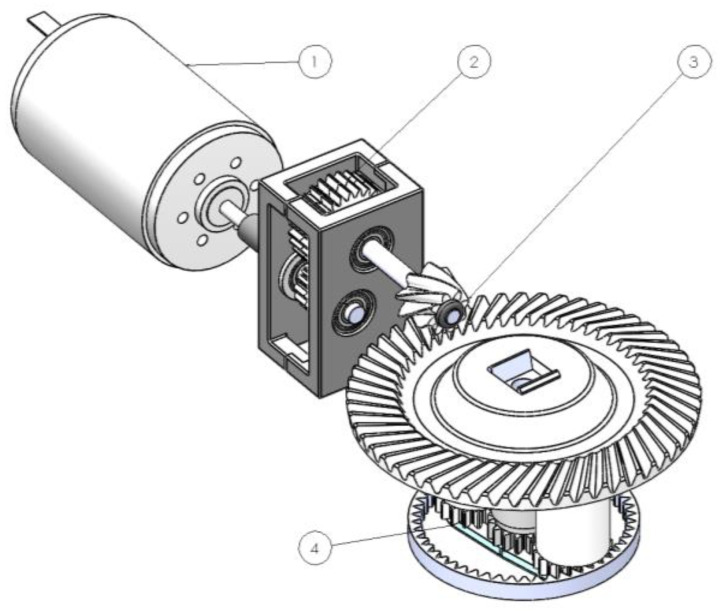
Mechanism design layout. The power is generated from the motor (1) and is transmitted directly to the first stage (2) consisting of a helical gear pair. The second stage features bevel gears (3) which translate the motion vertically, to be transmitted to the planetary gearset (4) which in turn compresses the tube.

This section focuses on the CAD modelling and the simulations of the mechanism. The total assembly is illustrated in Figure 1.

As depicted in Figure 1, the motion is generated by the motor and is directly driven to the two reduction stages of the powertrain. The powertrain connects to the planetary gearset which houses two planet gears, both of which are integrated at the bottom part of the rollers which compress the tube transporting the fluid. The connection of the two parts is performed through a snap-fit mechanism, thus offering stability while also providing a seamless way to install and assemble the mechanism. Notably, the employment of snap-fit mechanisms is a prominent approach to assemble injection-molded components, particularly within smaller mechanisms where spatial constraints are present and tolerances are tight. The key requirements of snap-fit mechanisms are strength, constraint, compatibility, and robustness [49].

**Figure 2:**
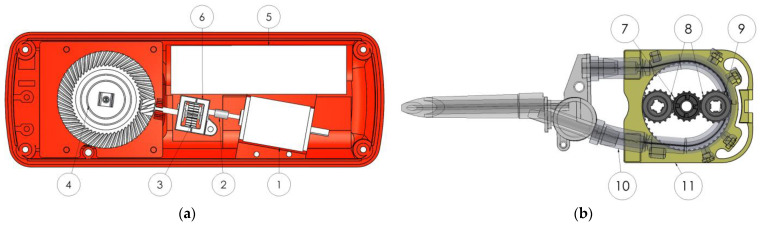
Powertrain layout. (**a**) The fixed part of the mechanism (in red) is the housing. On the bottom right, the motor is visible (1), which transfers the motion through an elastic coupling (2) to the helical gear pair of the first stage (3). This in turn translates the motion to the bevel gear pair of the second stage (4). The driven bevel gear features a snap-fit mechanism on the output shaft. On the top right, the battery is shown (5). The first stage has a separate housing (6). On the other side of the mechanism there is the planetary system which leads to the tube. (**b**) Design layout of the planetary gearset. In the middle of the planetary gearset there is the sun (7) which transfers the motion to the planets (8). The planets drive the rollers (9) which in turn compress the tube (10). The outer part is the housing (11).

Formulating a comprehensive and precise CAD model not only lays the groundwork for a more trustworthy simulation within the KISSsoft/KISSsys software package, but also contributes to an enriched range of attainable optimized geometries, which will significantly contribute to the optimal design selection based on the aforementioned criteria. The design approach incorporates a helical gear pair for the first stage, and a bevel gear pair for the second one. The output of the powertrain leads to the disposable part of the pump unit, that bears the planetary stage. This stage is responsible for the motion of the rollers, which compress the tube and, in turn, transport the fluid. The arrangement of the stages is demonstrated in Figure 2, as designed in the CAD model.

#### 2.1.2. Simulation Model Setup

**Figure 3:**
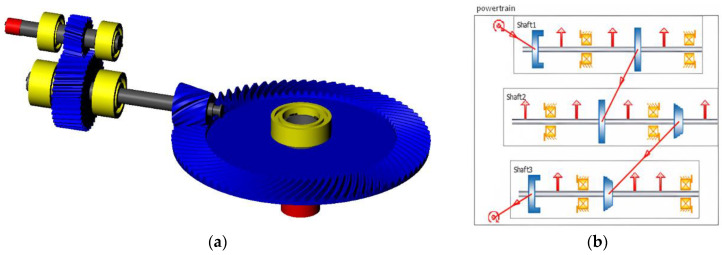

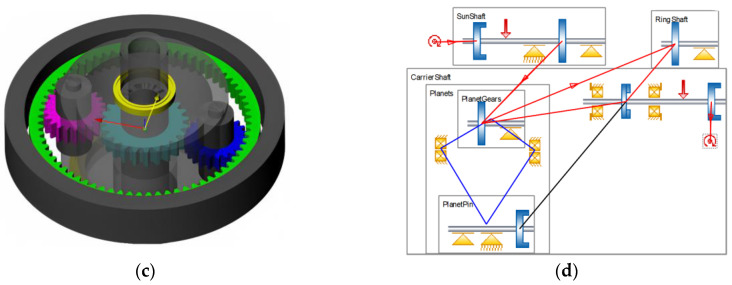
Simulation of the mechanism in the KISSsoft/KISSsys environment: (**a**) Model of the powertrain; (**b**) power flow of the system. Motion is entered in Shaft 1 (input shaft) and is transmitted through the helical gear pair to Shaft 2 (counter shaft). The bevel gear pair transmits the motion to Shaft 3 (output shaft), where it exits towards the planetary system of the pump; (**c**) model of the planetary gearset; (**d**) power flow of the system. The motion enters the subsystem through the sun shaft, which transmits it to the planet gear which finally connects to the ring.

The geometry is simulated using the KISSsoft and KISSsys software package. Exiting the powertrain, the power enters the planetary gearset via the snap-fit mechanism. The motion is then transmitted to the rollers compressing the tube and transporting the fluid. The models to be optimized are illustrated in Figure 3.

The simulation of the mechanism as an assembly was conducted in the KISSsys environment, while the characteristics of the machine elements that are used in each case were selected and examined in KISSsoft. Therefore, parameters such as the total efficiency and topological arrangement of each mechanism were calculated in KISSsys, while gear mesh efficiency, noise comparison, and gear stresses were calculated in KISSsoft.

### 2.2. Material Selection

For this study, the materials that were considered for the construction of the gears were steel and thermoplastic materials. In general, non-metallic gears have about 10 times less load capacities compared to their metallic counterparts [50]. Steel gears are far more dependent on the manufacturing accuracy to ensure silent and efficient operation. This claim is supported by the difference of the Young’s modulus between thermoplastic materials and steels that range in tens of orders of magnitude. This vast difference in compliance allows plastic gears to be less prone to load sharing and dynamic phenomena such as rattling at the expense of efficiency (versus lubricated steel gear pairs). Since no lubrication can be used due to the restrictions of the application at hand, steel and thermoplastic materials demonstrate similar friction coefficients. Furthermore, manufacturing of steel gears in the micro-scale can rarely be performed with traditional high-productivity methods such as hobbing, whereas plastic gears can be massively produced using injection molding techniques. Finally, the load range of the geartrain is relatively low allowing for the use of plastic gears.

Steel gears need to be lubricated often to ensure normal operation which, due to the nature of the application, would be a difficult endeavor. There are, however, ways to overcome this difficulty, such as using self-lubrication systems using specialized lubricants. Ebner et al. studied the operating behavior of self-lubricating gears based on oil-impregnated sintered material [51]. A simpler method would be to incorporate and inject lubricant from inside a reservoir located inside the case. Steel gears would, however, still not be ideal, since any type of lubrication would present a cross-contamination risk with the working fluid of the peristaltic pump.

On the other hand, non-metallic materials and especially thermoplastic materials present some useful characteristics. Since lubrication is prohibited, their frictional behavior provides a significant advantage by providing the option for dry run gear meshing. In addition, plastic gears offer lower noise levels, filling an important parameter of this research. All of the above can be achieved at a very low cost per part, since thermoplastic gears can be constructed with good accuracy using injection molding. On the other hand, they have lower durability than steel gears as well as lower conductivity, raising concerns for overheating [50].

Suitable materials for this application include a wide range of thermoplastic materials which can be additively manufactured, since this method allows the production of parts that have almost no geometry restrictions, even at a microscale [52]. The most commonly used thermoplastic materials are POM (polyacetal, such as POM-C) and PA (polyamide, such as Nylon 66) [50]. Both materials are suitable for this application due to their similar characteristics. The data in this research indicate that Nylon 66 has better frictional behavior than POM, and, therefore, provides an advantage regarding higher efficiency under dry running conditions. PA66 has slightly higher thermal conductivity values, thus it is safer regarding overheating. Therefore, the material that was selected to run the simulation was PA66. The material parameters are presented in Table 2, taken from [53].

### 2.3. Computational and Optimization Methods

The optimal design metrics for this research are high efficiency, low noise levels, high service life, and a suitable size. This section presents a comprehensive analysis of the methods as computed by the software [54,55], alongside analytical methods that have been employed in order to model and optimize the system.

#### 2.3.1. Computational Methods and Governing Equations

The efficiency calculation is presented in accordance to the ISO/TR 14179 standard, Part 1 and Part 2 [56,57], which calculates the heat level in a particular gearing unit. This study uses Part 2 [57], which calculates the heat transfer coefficient through an approximation of the shape of the housing in order to calculate heat dissipation. Meshing losses are taken into account for gears, while losses dissipated from rolling and sliding friction are considered for bearings.

The reliability calculation is based on Bertche’s study which recommends the use of the 3-parameter Weibull distribution as the preferred calculation method [58]. The reliability R of a machine element is calculated from (1):

(1) $$R(t)\;=\;e^{-{\frac{t-t_{0}}{\left(T-t_{0}\right)}}^{\beta_{w}}}\times 100\%$$

This equation uses the number of load cycles t, the Weibull parameter $\beta_{w}$, and the parameters T and $t_{0}$ which are derived from the mathematically achievable service life of the component, as demonstrated by the equations in the KISSsoft/KISSsys 2022 software manual [45].

The transmission error and tooth stresses are calculated through the contact analysis module of the KISSsoft software. Contact analysis is an iterative method. For this study, a high level of accuracy was used in order to ensure the accuracy of calculation, corresponding to a $\varepsilon =10^{-5}$ termination criterion. The convergence condition is shown in Equation (2), where $T_{c}$ the calculated torque and $T_{n}$ the nominal torque.

(2) $$\left|\frac{T_{c}}{T_{n}}-1\right|\leq \varepsilon$$

In the specific context of the planetary gearset’s contact analysis model, the planet carrier revolves around a stationary sun gear and an inner (ring) gear. Each of the planets utilize the stiffnesses between the sun/planet and planet/internal gear pairs to adjust its rotational position, effectively offsetting all torques. This method requires an iterative computation of the system to align the sun’s torque with the designated nominal torque.

In this case, the transmission error is calculated as a length on the path of contact in μm. The transmission error is defined according to [59] for helical toothed gears. It is noted that the PPTE is an important factor in noise generation during operation.

The formulae in the standard [59] were utilized in order to determine the tooth root stresses in the segments. KISSsoft applies a “graphical method”, which offers an enhancement in functionality compared to the standard. This method applies the stress calculation process using the formulae from the standard to the cross-sections in the range of the 30° tangent, not just at the 30° point. Thus, it calculates the cross-section (diameter) at the point on the tooth at which the maximum tooth root stress is located. This also makes use of the formulae in the standard for the relevant cross-section [45].

#### 2.3.2. Optimization Approach

The adopted approach consists of two discrete phases; the optimization and the tailored optimal design selection. The first phase utilizes the fine sizing tool of the KISSsoft software in order to internally optimize the machine elements, as analyzed by the KISSsoft AG demonstration [60]. This calculates and displays all possible gear geometries for some predefined characteristics, such as facewidth and center distance (gear rim diameter for planetary stages) [45]. Turci [61] defines the optimization in KISSsoft as an activity, during which the software conducts design optimization algorithms by relying on three main concepts: objectives, constraints, and variables. After establishing these three concepts, a wide range of options is generated, and the optimal solutions are displayed based on clearly defined criteria [61]. An approach on the use of KISSsoft optimization algorithms was first applied to beveloid gears [62], but paves the way for other gear types as well. In the studies [61,63], it was demonstrated how rapidly macro-geometry variations can be produced using established software suites. A similar concept was applied for generating micro-geometry variations, as outlined in one of AGMA’s Fall Technical Meetings (FTM) [61,64]. These contribute to the presentation of an internal multi-objective optimization [61]. Can et al. [65] have also used the software in order to conduct an optimization of gear geometrical parameters, carried out under three set constraints. The authors consequently discuss and examine the results yielded by the software and select the final geometries.

**Figure 4:**
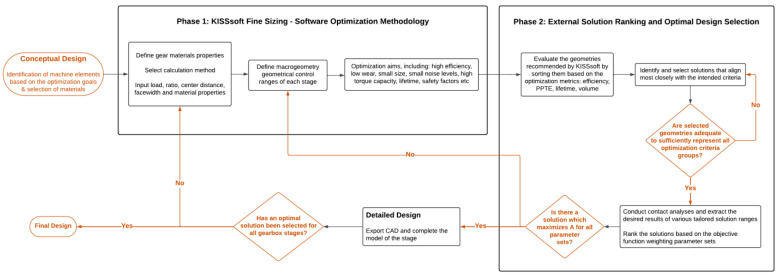
Layout process of gearset optimization and optimal design selection.

The second phase concerns the external ranking and the optimal design selection based on the selected criteria and weighting parameter sets following the optimization conducted by the software. This is achieved by externally using the objective function and evaluating an extensive range of geometries, as yielded by the optimization algorithm of KISSsoft in the first phase. Some of these geometries have been distinguished and selected for further investigation as presented in this work. It should be noted that the analyses were conducted according to VDI 2736 for thermoplastic materials [45]. The selected optimization criteria that affect the optimal design selection include the efficiency, volume, PPTE, and reliability. The optimization and optimal design selection methodology is outlined in Figure 4, alongside the broader methodology being employed.

An objective function was constructed to rank a range of the optimized geometries calculated by KISSsoft during the subsequent optimal design process. The considered factors are normalized in each case, receiving values from 0 to 1. This range is determined for each parameter based on the following normalization relationship, where *X* is the respective parameter, based on the upper and lower set boundaries, as shown in Equation (3):

(3) $$\mathrm{norm}(X)\;=\;\frac{X-lower\left(X\right)}{\mathrm{upper}\left(X\right)-lower(X)}$$

Normalization is either performed based on a maximum (upper boundary) and minimum (lower boundary) value of each factor among the solutions, or by artificial limits, which are placed based on the system specifications when a metric is fulfilled by a particular set boundary. It is stated that for the volume and noise parameters, the 1 − *norm*(*X*) coefficient is considered due to the need to minimize these values. The weighting parameters attributed to each factor of the objective function are presented in Equation (4). The aim is to maximize the value *A* of the objective function:

*A* = *α*_1_
*norm*(*η*) *+ α*_2_
*norm*(*L*) *+ α*_3_ [1 − *norm*(*N*)] *+ α*_4_ [1 − *norm*(*V*)] (4)

where η is the efficiency, L is the service life, N is the PPTE, and V is the volume of each solution. Note that the parameter $\alpha_{i}$ represents the weight given to each individual criterion. The value for each parameter is displayed indicatively in Table 3 for four different cases. These parameters are to be used to determine the value of the objective function, based on the requirements of each individual case.

## 3. Results and Discussion

This section demonstrates the results obtained from the simulations of the model. The evaluation delves into the optimization and optimal design selection parameters which have been examined for each powertrain stage, as well as for the planetary gearset. Various geometries were studied and the associated results have been visualized. These geometries were ranked using the objective function presented in Equation (4). The results of the optimization metrics output by the selected geometries were then inspected regarding their integrity.

### 3.1. Optimal Design Selection

A range of geometries were examined for each individual stage, to be ranked using the objective function presented in Equation (4). Each parameter takes control ranges in which possible geometries are explored, divided for each stage of the gearbox. Table 4 showcases the control ranges for the center distance (*a*), normal pressure angle ($\alpha_{n}$), facewidth (*b*), helix/spiral angle (*β*), normal module ($m_{n}$), and number of teeth (*Z*).

**Figure 5:**
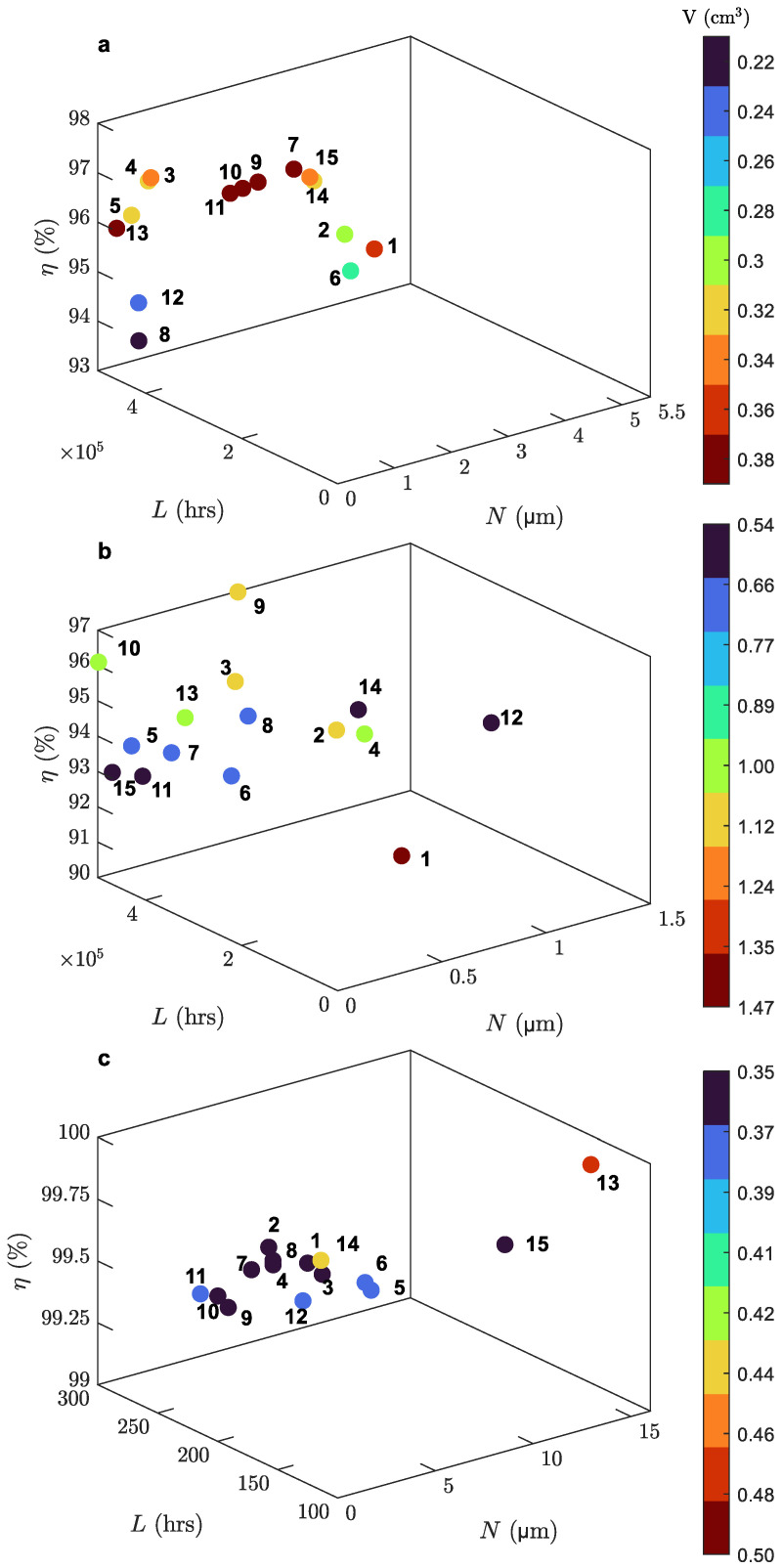
Pareto fronts for each stage: (**a**) first stage; (**b**) second stage; (**c**) planetary gearset.

**Figure 6:**
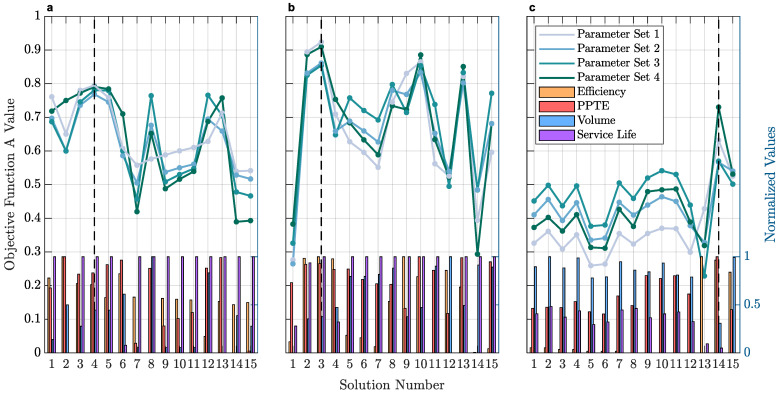
Objective function values as yielded by the objective function in the second optimization phase for the: (**a**) first stage; (**b**) second stage; (**c**) planetary gearset.

According to the conducted KISSsoft optimization, the Pareto fronts of Figure 5 were constructed to visualize the optimized geometric parameter combinations yielded by KISSsoft. Based on these control ranges and given the objective function of Equation (4), the optimal solutions for each stage were explored. A total of 15 solutions for each gearbox stage were systematically evaluated based on the KISSsoft fine sizing results of the first optimization phase. Consequently, in the second optimization phase, these solutions were ranked using the objective function in order to determine each stage’s optimal design. Different solutions may produce comparable results, a consideration addressed by the employment of the distinct objective function parameter sets. All the objective function parameter sets for both the powertrain and the planetary gearset were evaluated accordingly. Figure 6 displays the objective function *A* values alongside the respective nondimensionalized metric bar values, that have been produced as a result of ranking the candidate optimal design solutions via the objective function.

The geometries that yield the most prominent combination of results for each stage were determined by the Pareto fronts and their corresponding objective function graphs to be solution #4, solution #3, and solution #14 for the two powertrain stages and the planetary gearbox, respectively. Table 5 showcases the center distance (*a*), normal pressure angle ($\alpha_{n}$), facewidth (*b*), helix/spiral angle (*β*), the diameters (*D*), the gear mesh contact ratios ($\varepsilon_{c}$), the normal module ($m_{n}$), the profile shift coefficient (*x*), and the number of teeth (*Z*) for these selected designs after the second phase of the optimization process.

### 3.2. Results of Optimized Design Solution Contact Analyses

**Figure 7:**
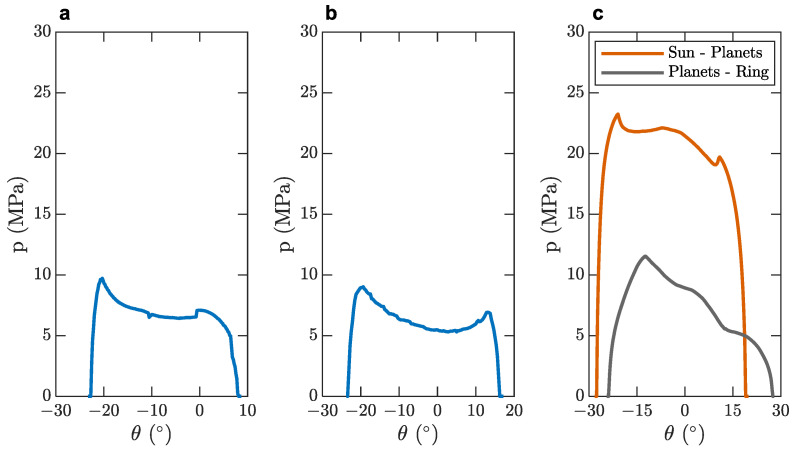
Gear Hertzian stresses: (**a**) first stage; (**b**) second stage; (**c**) planetary gearset.

The Hertzian stresses received by the gears are a critical quantity and thus they were monitored through the simulation. Figure 7 displays, for each gearbox stage, the Hertzian stresses of the selected optimized design, as demonstrated in Table 5, following the second phase of the optimization process.

The trends of the graphics displayed in Figure 7 correspond to the expected forms along the path of contact, as studied by [66,67]. Each graph refers to the Hertzian stresses, which coincide with each other for both the driving and driven gears per contact pair. The Hertzian stresses on the sun–planet contact pair exhibit significantly higher values compared to the first and second reduction stages, as well as the planet–ring contact pair. As far as the planet–ring contact pair is concerned, the contact stress reduction is attributed to the internal (concave) gear geometry that significantly reduces contact stresses in accordance with both the Hertzian theory and the ISO 6336-2 [68] calculation method. The planetary stage is disposable, as previously demonstrated by the respective service life specifications of Table 1. Therefore, the high contact stresses it receives are deemed acceptable.

**Figure 8:**
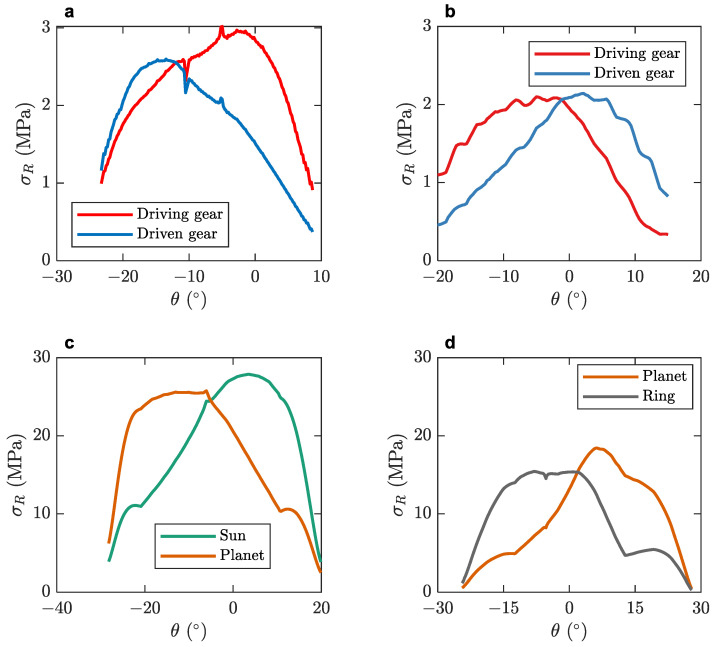
Gear tooth root stresses: (**a**) first stage; (**b**) second stage; (**c**) sun–planets; (**d**) planets–ring.

Respectively, more information about the root stresses that occur in the tooth root area using the graphical method are provided in Figure 8 where the fluctuation of the maximum principal stress is calculated along the path of contact of the meshing gears, according to VDI 2736 [45,59]. According to [45], the VDI 2736 method includes the empirical, tooth root, tooth flank, deformation, and wear calculations as described in [59]. Figure 8 illustrates the tooth root stresses. The focus of both Figure 7 and Figure 8 lies in testing the gears durability under the intended operation conditions.

The stress distribution against the angle of rotation of Figure 8 displays a similar trend as the one studied in Ref. [66]. The area of the central root stress is located near the maximum stress point on the graphs [69].

The planetary gearset in Figure 8 receives higher stresses due to the higher torque levels received in that area following the first two reduction stages. As discussed in [70], planetary gearsets are, in general, more fit to withstand higher power densities and gear ratios, which leads to higher torque and thus stress levels. Since the planetary stage is disposable, the high tooth root stresses are also deemed acceptable.

As demonstrated by the graphs in Figure 7 and Figure 8, the maximum stresses that the gears receive when operating are lower than the yield strength of PA 66, which is over 60 MPa, as demonstrated in [71]. This affirms the gears’ robust performance in a real-world scenario since the safety factor is greater than 2.

**Figure 9:**
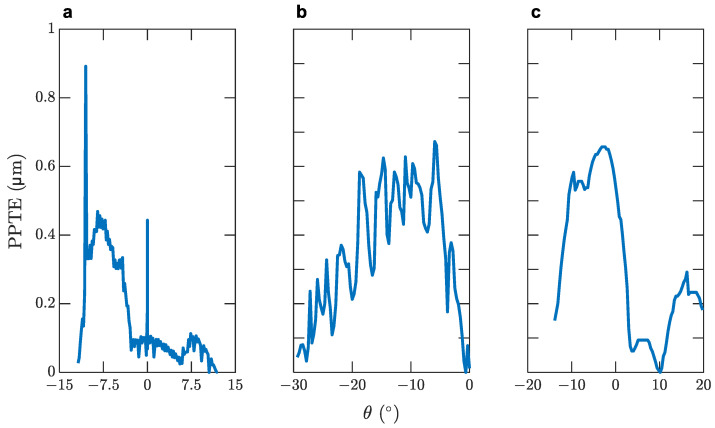
Transmission error for the: (**a**) first stage; (**b**) second stage; (**c**) planetary gearset.

These led the investigation to the transmission error produced by the gears when operating. Figure 9 shows the model’s peak-to-peak transmission error as induced by the optimal solutions yielded by the objective function. The transmission error is calculated along the path of contact under load. The diagrams in Figure 9 showcase the displacement of the contact point of the driving gear as a function of the angle of rotation of the driven gear, shifted towards the axis’s origin. Apart from the relationship of the PPTE with noise generation, the steepness of the slopes and, therefore, the acceleration of the transmission error should not be ignored since high acceleration produces additional loads, which may have a significant effect on noise generation [45].

The results indicate that the transmission error and, therefore, the rattling noise of the gears, is constrained in low values compared to other solutions that have been examined using KISSsoft. By employing an increased helix angle to the gears, an elastic averaging of errors is created and the PPTE is reduced [42], a statement that is in accordance with the results of the simulations. The helix angle incorporated in the gears of the planetary system contribute to an overall reduced PPTE, which is particularly significant in medical equipment applications [72]. In the case of planetary gearsets, studies have shown that a larger number of planets improve the load sharing conditions since the operation load is distributed to multiple planets [73,74]. However, this conclusion can be supported only by theoretical models since, in reality, a larger number of planets would increase the sensitivity to errors [74]. In this particular model, since the planets are inseparably attached to the rollers that control the fluid’s flow rate, the use of two planets is mandated. It is noted that the selected optimal solution for the planetary gearset displays low mechanical noise when compared to other examined candidates.

The main optimization parameters of the selected optimal design in the form of the efficiency (*η*), peak-to-peak transmission error (*Ν*), volume (*V*), and service life (*L*) of the optimized design are summarized in Table 6. The results are presented for the chosen optimized geometry parameters in Table 5.

These findings relate to theoretical gear mesh properties, which would vary in a real-world operational setting. This provides the opportunity for experimental validation, a crucial step towards achieving a more comprehensive and practically oriented optimal design of the subject. This theoretical model, using a helical and bevel gear pair for the first and second stage, respectively, showcases high efficiency, positioning itself as an optimal choice for scenarios with an emphasis on energy conservation. It also demonstrates commendable levels of reliability, coupled with comparable volume considerations, rendering them contenders adaptable to a range of engineering requisites.

The geometry selected for the planetary gearset also shows excellent levels of gear mesh efficiency, demonstrating operation with minimal losses. The service life of the disposable part is also, as expected, quite low when compared to the powertrain counterpart. This part is designed to be replaced every few operation cycles and as such a service life of 124.66 h is deemed satisfactory. The peak-to-peak transmission error is also higher than the one evaluated for the second stage of the powertrain, which is attributed to the fact that planetary gearsets tend to be volatile to complexities when it comes to meshing, as well as misalignments and manufacturing tolerances.

## 4. Conclusions

This research delved into the underexplored area of investigating the driving mechanisms of micro- and meso-scale peristaltic pumps. The gearbox is comprised of two key mechanisms: the powertrain and the planetary gearbox. Both have been thoroughly examined to optimize their operational principles. This process aims to achieve four simultaneous goals: maximize system efficiency and reliability while concurrently minimizing transmission error and volume. A thorough examination of the candidate machine elements has been explored in order to identify the most suitable ones that meet the needs of this application. Consequently, the main objective of this work lies in the comprehensive optimization of the system. This includes refining the selected individual components, which plays a pivotal role in shaping concise and credible simulation results. To ensure this, the next step was to employ specialized software for further analysis. To this end, the KISSsoft/KISSsys software package was utilized, which enables the assessment and modeling of a wide array of geometries, with specific ones being chosen for further investigation. The method of determining which ones are chosen depends on the four aforementioned optimization parameters. These distinguished solutions were ranked using the formulated objective function for four distinct scenarios, each characterized by a unique set of weighting parameters. Visual representations of the resulting geometries, including Pareto fronts and weighting graphs, facilitated the identification of an optimal configuration for each of the two powertrain stages and for the planetary gearset. These findings were subsequently subjected to further simulation, focusing on a comprehensive stress analysis, which revealed the endurance of the gears composing the gearbox. The optimum designs have ultimately culminated in a commendable combination of gear mesh efficiency, PPTE, volume, and service life for the examined components.

In light of the promising results obtained in this study, future research endeavors may extend into experimental validation, providing data to confirm the simulation outcomes. Experimental setups could be devised to also examine the performance of the peristaltic pump, as well as its driving mechanisms. Furthermore, considering the critical role of the fluid–structure interaction in the operation of the pump, future investigations may delve into in-depth FSI simulations. This would offer a comprehensive understanding of the flow rate and fluid dynamics in effect, paving the way for even more refined design optimizations.

## Figures and Tables

**Table 1:** Peristaltic pump operating technical specifications.

	First Powertrain Stage	Second Powertrain Stage	Planetary Gearset
Transmission ratio	2 ± 5%	6.75 ± 5%	4.25 ± 5%
Input speed (rpm)	2000	1000	148
Output speed (rpm)	1000	148	34
Input torque (Nmm)	2.4	4.8	32.4
Output torque (Nmm)	4.8	32.4	137.7
Minimum service life (h)	20,000		100

**Table 2:** Material properties of PA66 [53].

Density (g/cm^3^)	1.39–1.42
Modulus of elasticity (tensile), *E* (MPa)	2600–3200
Tensile strength (MPa)	60
Yield Strength (MPa)	67
Elongation at yield (%)	9
Poisson’s ratio, *v*	0.42–0.45
Service temperature (short term) (°C)	110–140
Service temperature (long term) (°C)	90–00
Thermal expansion, *α* (K^−1^)	13–14 × 10^−5^
Thermal conductivity, *λ* (W/K∙m)	0.2–0.39
Specific heat, *c* (J/g∙K)	1.4
Friction coefficient for same material combination	0.2

**Table 3:** Values of the weighting parameters used in the ranking objective function for four indicative cases.

$\mathrm{Parameter}\;a_{1}$	$\mathrm{Parameter}\;a_{2}$	$\mathrm{Parameter}\;a_{3}$	$\mathrm{Parameter}\;a_{4}$
0.4	0.3	0.2	0.1
0.4	0.3	0.1	0.2
0.3	0.3	0.1	0.3
0.3	0.4	0.1	0.2

**Table 4:** Parameter control ranges for the first stage (helical gear pair), the second stage (bevel gear pair), and the planetary gearset.

Parameter	First Stage	Second Stage	Planetary Gearset
*a* (mm)	6–8	–	4.10–8.60
$a_{n}$ (°)	20	20	20
*b_driving_* (mm)	2.5–3	4–5.5	2
*b_driven_* (mm)	3–4	4–5.5	2
β (°)	0–40	0–40	0–16
$m_{n}$ (mm)	0.2–0.4	0.3–0.5	0.3–0.5
*Z_driving_*	14–21	8–12	–
*Z_driven_*	26–40	54–81	–
*Z_p_*	–	–	15–26
*Z_r_*	–	–	50–70
*Z_s_*	–	–	13–22

**Table 5:** Optimized parameters of the chosen solutions for the three gearbox stages.

Magnitude	First Stage		Second Stage		Planetary Gearset			
	Driving	Driven	Driving	Driven	Sun	Planets		Ring
*a* (mm)	7.5		-		6.50		–	
$a_{n}$ (°)	20		20		20			
b (mm)	3	4	5	5	2			
β (°)	20		39		16			
$D_{inner}$ (mm)	2	2	2	5	2.67	4.14		-
$D_{root}$ (mm)	4.07	9.28	4.12	35.32	4.10	6.97		21.91
$D_{pitch}$ (mm)	4.78	9.57	4.68	36.34	4.72	7.64		20.76
$D_{tip}$ (mm)	5.42	10.58	5.86	-	5.71	8.58		20.28
$D_{outer}$(mm)	5.42	10.58	5.86	34.17	5.71	8.58		24.89
$\varepsilon_{c}$	1.089		1.157		–			
$\varepsilon_{c,s-p}$	–		–		1.140		–	
$\varepsilon_{c,p-r}$	–		–		–		1.466	
$m_{n}$ (mm)	0.3		0.3		0.35			
x	0.376	0.817	0.44	−0.44	0.54	0.481		−0.331
Z	15	30	12	81	13	21		57

**Table 6:** Summarized optimal design parameters for the powertrain.

Optimization Parameter	First Stage	Second Stage	Planetary Gearset
η (%)	96.55	96.98	99.5
N (μm)	0.87	0.0916	0.657
V (cm^3^)	0.31	1.12	0.435
L (h)	>500,000	253,123	124.66

## Data Availability

Data is contained within the article.

## Nomenclature

A	Objective function value
*a*	Center distance (mm)
*b*	Facewidth (mm)
D	Diameter (mm)
L	Service life (h)
$m_{n}$	Normal module (mm)
N	Peak-to-peak transmission error (μm)
R	Reliability (%)
t	Number of load cycles
$T,t_{0}$	Reliability calculation parameters
$T_{c}$	Calculated torque (Nm)
$T_{n}$	Nominal torque (Nm)
V	Volume (cm^3^)
X	Objective function parameter
x	Profile shift coefficient
*Z*	Number of gear teeth
Greek letters	
$\alpha_{1},\alpha_{2},\alpha_{3},\alpha_{4}$	Objective function weighting parameters
$a_{n}$	Normal pressure angle (°)
β	Helix/spiral angle (°)
$\beta_{w}$	Weibull shape parameter
ε	Value of termination criterion
$\varepsilon_{c}$	Gear mesh contact ratio
η	Gear mesh efficiency (%)
θ	Angle of rotation (deg)
$\sigma_{R}$	Tooth root stress (MPa)
*p*	Hertzian stress (MPa)
Subscripts	
driven	Driven gear
driving	Driving gear
root	Gear foot circle
inner	Gear inner circle
tip	Gear tip circle
pitch	Gear pitch circle
p	Planetary gearset planets
r	Planetary gearset ring gear
s	Planetary gearset sun gear
Abbreviations	
CAD	Computer Aided Design
CFD	Computational Fluid Dynamics
DC	Direct current
FSI	Fluid–structure interaction
HPPSCDD	Hydraulic-driven piezoelectric pump with separable channel for drug delivery
MEMS	Micro-electromechanical systems
PA	Polyamide
PMP	Peristaltic micropump
POM	Polyoxymethylene
PPTE	Peak-to-peak transmission error
